# Characterization of Microbial Community and Flavor Compounds in Traditional Fermented *Douchi* Using HTS and HS‐SPME‐GC–MS

**DOI:** 10.1002/fsn3.4660

**Published:** 2024-12-19

**Authors:** Aiguo Luo, Tiantian Liu, Shengli Shi, Xiaoxia Liu, Xiaoli Shi, Bianfang Hu

**Affiliations:** ^1^ Department of Biological Science and Technology Jinzhong University Jinzhong China; ^2^ Shanxi Center of Technology Innovation for Compound Condiment Jinzhong University Jinzhong China; ^3^ Jinzhong Normal College Jinzhong China

**Keywords:** characteristic flavors, correlation analysis, *Douchi*, microflora community structure

## Abstract

This study aim is to elucidate the relationship between the microbial community dynamics and the production of volatile flavor compounds during the fermentation process of bacterial‐type *Douch*i. Using high‐throughput sequencing (HTS) and headspace solid‐phase microextraction, gas chromatography–mass spectrometry (HS‐SPME‐GC–MS) was used to investigate microbial diversity and volatile compound profiles at different fermentation stages. Spearman correlation analysis was employed to identify potential associations between microbial genera and flavor compounds. The results revealed that the dominant bacterial phyla were Pseudomonadota and Firmicutes, with the dominant genera being *Bacillus*, *Enterobacter*, and *Weissella*. The dominant fungal phyla were *Ascomycetes* and *Mucoromycota*, whereas the dominant genera were *Aspergillus*, *Trastula*, and *Pythium*. A total of 110 volatile substances (relative to a relative content ≥ 0.03%) were detected during the fermentation process of bacterial‐type *Douchi*. These included 34 hydrocarbons, 17 alcohols, 11 heterocyclic compound, 10 acids, 9 ketones, 9 esters, 6 aldehydes, 6 sulfur compounds, and 8 other compounds. The characteristic flavor compounds identified were ethanolamine, 2,3‐butanediol, benzoaldehyde, 3‐hydroxy‐2‐butanone, pterin‐6‐carboxylic acid, ethyl heptanoate, and diallyl disulfide. Correlation analysis indicated a strong positive association between benzoin aldehyde and pterin‐6‐carboxylic acid with the genera *Thiobacillus* and *Pythium*. Ethyl heptanoate was positively correlated with *Enterobacter*, *Weissella*, and *Trasmium*. Additionally, 3‐hydroxy‐2‐butanone was positively correlated with the genus *Staphylococcus*. This research provides valuable insights into the microbial and chemical dynamics of bacterial‐type *Douchi* fermentation, offering guidance for optimizing fermentation processes to improve product quality.

## Graphical abstract

This study investigates the microbial community dynamics and volatile flavor compounds during the fermentation of traditional Douchi using high‐throughput sequencing (HTS) and HS‐SPME‐GC‐MS. The results reveal significant correlations between specific microbial genera and flavor substances, providing insights into optimizing fermentation processes to enhance product quality.

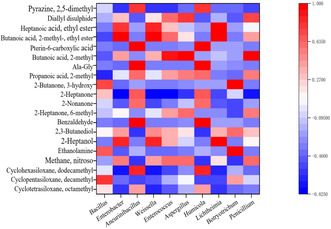

## 1 Introduction

*Douchi*, a traditional Chinese fermented product with origins dating back to the Han Dynasty, is renowned for its distinctive aroma and rich flavor, making it highly favored by consumers (Wang et al. 2021). Based on the different microorganisms involved in fermentation, *Douchi* can be classified into four categories: bacterial type, Aspergillus type, Mucor type, and Rhizopus‐type (Xu et al. 2022). The common principle underlying the fermentation process in all four types involves the hydrolysis of soybeans into small peptides and amino acids through microbial activity (Zhang et al. 2023). This process also produces various bioactive substances beneficial to human health, such as soybean isoflavones and *Douchi* Plasmin). These small peptides, secondary products, and metabolites undergo complex biochemical reactions during fermentation, including decarboxylation and esterification, contributing to the unique flavor of *Douchi* (Liu et al. 2020). Thus, the composition of the microbial community during fermentation plays a significantly role in determining the flavor and quality of *Douchi*.

The most typical form of bacterial‐type *Douchi* in China is Sichuan wet‐*Douchi*, whose production process primarily involves four steps: soaking, steaming, koji making, and fermentation (Huang et al. 2020). Among these steps, koji making and fermentation are the most critical. Liang Yexing (Liang et al. 2017) noted that the microorganisms involved in the koji making and fermentation of bacterial‐type fermented soybeans mainly include *Bacillus subtilis* , lactic acid bacteria, and *Micrococcus*. Any changes in fermentation conditions can lead to alterations in the microbial community structure. As a result, researchers have conducted extensive studies on the microbial community during the fermentation process of *Douchi*. For instance, Li, Liu, and Zhang (2014) and Li et al. (2020) used high‐throughput sequencing (HTS) technology to explore the effects of different factors on microbial diversity in *Douchi*. Li et al. (2024), Yang, Tan, and Zhou (2023), and others incorporated sensory evaluation and OCV analysis to determine the optimal production process of *Douchi* based on changes in the microbial community. However, microorganisms at different stages of fermentation stages may play distinct roles in flavor formation, and variations in microbial composition significantly affect the flavor of *Douchi*. This study focuses on the natural fermentation process of bacterial‐type *Douchi*. By employing HTS and HS‐SPME‐GC‐MS technologies, this study analyzes the changes in microbial community structure, volatile flavor substances, and physicochemical properties during the fermentation process. Spearman correlation analysis was used to investigate the relationship between microbial community structure and characteristic flavor compounds. This study provides a valuable reference for understanding the correlation between microbial community structure and characteristic flavor.

## 2 Materials and Methods

### 2.1 Bacterial‐Type *Douchi* Fermentation and Sampling

Bacterial‐type *Douchi* was produced using a natural fermentation method. The raw materials included soybeans (15 kg), salt (1 kg), Baijiu (1 L), chili (500 g), ginger (1 kg), and garlic (1 kg), all purchased from the Shanxi Farmers' Market in China.

A total of 1.5 kg of mature, undamaged soybeans was selected, cleaned, and soaked for 24 h. The soaked soybeans were then cooked at 100°C for 3 h until they became soft and easily crushable. The cooked soybeans were divided into five groups of 300 g each. These groups were placed in gauze bags at 45°C to regulate moisture content and then transferred to an insulated box. A bamboo steamer was placed at the bottom to facilitate natural fermentation at 28°C, ensuring moisture retention and breathability throughout the process. Additionally, 1 L of boiled soybean water and 5 g of salt were stored at 4°C for later use. After 72 h of fermentation, white mycelium growth was observed. Subsequently, 10 g of salt, 20 mL of Baijiu, 10 g of pepper, 15 g of shredded ginger, and 15 g of garlic puree were added to each group, mixed thoroughly, and placed in fermentation tanks. The prepared soybean water was added to submerge the soybeans. The temperature of the fermentation tanks was maintained at 25°C for secondary fermentation. The bottles were sealed, and the product was ready for consumption after 15 days. Fermentation was conducted in five tanks, with three parallel replicates, resulting in a total of 45 tanks.

Samples were randomly collected on the 1st, 3rd, 5th, 9th, and 15th days of fermentation. A 2.0 g *Douchi* sample was cut into small pieces using high‐temperature sterilized scissors, mixed thoroughly, and 0.5 g was placed in a 1.0 mL sample tube, labeled as DC1G, DC3G, DC5G, DC9G, and DC15G. These samples were sent to Shanxi Saikunsi Biotechnology Co. Ltd. for high‐throughput sequencing (HTS) analysis. Similarly, 0.5 g of lobster sauce was placed in a 2.0 mL sample tube, labeled as DC1Q, DC3Q, DC5Q, DC9Q, and DC15Q, and sent to the Biological Detection Laboratory of Shanxi Agricultural University for headspace solid‐phase microextraction gas chromatography–mass spectrometry (HS‐SPME‐GC‐MS) analysis. Each sampling was performed in triplicate, resulting in a total of 45 samples for HTS and 45 samples for HS‐SPME‐GC‐MS analysis, totaling 90 samples.

### 2.2 Analysis of Microbial Community by High‐Throughput Sequencing Technology During Fermentation of Bacterial‐Type *Douchi*

#### 2.2.1 DNA Extraction

Total genomic DNA was extracted from 2.0 g of fermented *Dochi* samples using the E.Z.N.A. Soil DNA Kit (Omega Bio‐tek, Norcross, GA, USA). The extraction procedure followed the manufacturer's protocol with minor modifications. Samples were homogenized in a lysis buffer and incubated with Proteinase K at 56°C for 1 h. The lysate was then subjected to a series of centrifugation and washing steps to purify the DNA. The quality and quantity of the extracted DNA were assessed using a Nanodrop spectrophotometer and 1% agarose gel electrophoresis. The DNA samples were stored at −20°C until further analysis.

#### 2.2.2 PCR Amplification

The V3–V4 region of the bacterial 16S rRNA gene was amplified with forward primer 338F (5′‐ACTCCTACGGGGAGGCAGCA‐3′) and 806R (5′‐GGACTACHVGGGTWTCTAAT‐3′) (Edgar 2010). The ITS1 region of the fungal rRNA gene was amplified with forward primer ITS1F (5′‐GGAAGTAAAAGTCGTAACAAGG‐3′) and reverse primer ITS1R (5′‐GCTGCGTTCTTCATCGATGC‐3′) (Anderson and Willis 2003). The PCR reactions were performed in 25 μL volumes containing 12.5 μL of 2× PCR master mix, 1 μL of forward primer (10 μM), 1 μL of reverse primer (10 μM), 2 μL of DNA template, and 8.5 μL of nuclease‐free water (Anderson, Ellingsen, and McArdle 2006). The cycling conditions included an initial denaturation at 95°C for 3 min, followed by 30 cycles of 95°C for 30 s, 55°C for 30 s, and 72°C for 1 min, with a final extension at 72°C for 5 min (Anderson and Walsh 2013). All PCR reactions were performed in triplicate. The PCR products were verified by electrophoresis on a 1.5% agarose gel, and successful amplification was indicated by the presence of a distinct band of the expected size.

#### 2.2.3 Illumina Sequencing Analysis

The PCR products were purified using a QIAquick PCR Purification Kit (Qiagen, Germany) and quantified using a Qubit fluorometer. Sequencing libraries were prepared using the Nextera XT DNA Library Prep Kit (Illumina, San Diego, USA) by Shanghai Pai Senno Biological Technology Co. Ltd. The libraries were then sequenced on an Illumina MiSeq platform using the 2 × 300 bp paired‐end protocol. Raw sequencing data were processed using QIIME 2 for quality filtering, chimera removal, and taxonomic classification. Operational taxonomic units (OTUs) were clustered at a 97% similarity threshold, and representative sequences were aligned against the SILVA database for bacteria and the UNITE database for fungi.

### 2.3 Gas Chromatography–Mass Spectrometry Detection

Volatile flavor compounds in fermented *Dochi* were analyzed using headspace solid‐phase microextraction coupled with gas chromatography–mass spectrometry (HS‐SPME‐GC–MS) (GCMS‐QP 2010, Agilent Technologies Inc., USA). Two grams of the *Dochi* sample were placed in a 20 mL headspace vial, sealed, and preheated at 60°C for 10 min. A SPME fiber (50/30 μm DVB/CAR/PDMS, Supelco, USA) was exposed to the headspace at 60°C for 30 min to adsorb volatile compounds. The fiber was then desorbed in the GC–MS injection port at 250°C for 5 min (Geng and Li 2005). Chromatographic separation was performed on an Agilent DB‐5MS capillary column (30 m × 0.25 mm, 0.25 μm). The oven temperature program was as follows: initial temperature at 40°C for 3 min, ramped at 3°C/min to 150°C, held for 4 min, and then increased at 10°C/min to 240°C. The carrier gas was helium at a flow rate of 1 mL/min with a split ratio of 10:1. Mass spectrometry conditions included electron impact ionization (EI) at 70 eV, an ion source temperature of 230°C, and a mass scan range of 40–400 m/z. Volatile compounds were identified by comparing mass spectra with the NIST and Wiley libraries, with a match quality threshold of > 800. The relative content of each component was calculated using the peak area normalization method. This method provided a comprehensive profile of volatile compounds, essential for understanding the flavor characteristics of fermented *Dochi*.

### 2.4 Statistical Analysis

Each experiment was performed in triplicate. Results are expressed as mean ± standard deviation. Statistics analysis was conducted using SPSS software to evaluate the structure of microbial flora, its correlation with characteristic flavor, and to assess data variance, with a significance level of *p* < 0.05. HTS data were analyzed by clustering operational taxonomic unit (OTU). HS‐SPME‐GC–MS detected ion chromatograms, retrieved by computer libraries, and the components with similarity less than 90% were removed to identify volatile flavor components of *Douchi*. Origin 2022 software was used to generate heat maps, Veen plots, dilution curves, and relative abundances.

## 3 Results and Analysis

### 3.1 Sequence Richness and Diversity Analysis

**FIGURE 1:**
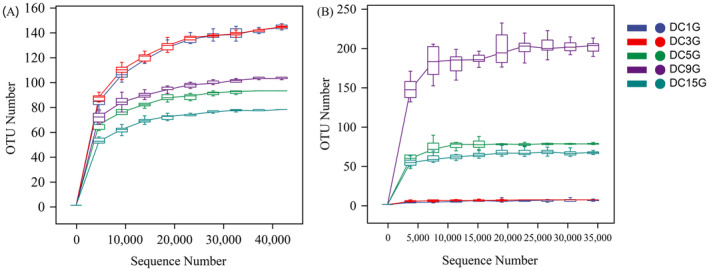
Dilution curve of *Douchi* sample. (A) Bacteria and (B) fungus.

Dilution curve analysis indicated significant variations in microbial diversity among the samples. In the bacterial rarefaction curves (Figure 1A), the OUT counts for samples DC3G and DC1G quickly reached saturation as sequencing depth increased. This indicated that these samples had relatively high bacterial diversity and that the sequencing depth was enough to capture most of this diversity. The DC9G sample also showed high bacterial diversity but reached saturation at a slightly greater sequencing depth, suggesting a need for deeper sequencing to fully capture its diversity. Conversely, the DC5G and DC15G samples reached saturation at lower sequencing depths, indicating lower bacterial diversity in these samples. For the fungal rarefaction curves (Figure 1B), samples DC1G and DC3G remained stable throughout the sequencing process, indicating a low number of fungal OTUs in these samples. For other samples, the OTU values tend to flatten out when the sequencing depth reaches 8000. All samples were ultimately plateaued, indicating that no additional bacteria or fungi species were detected with extended sequencing time. This suggests that the sequencing data are reasonable and accurately reflects the microbial community information in the *Douchi* samples.

**FIGURE 2:**
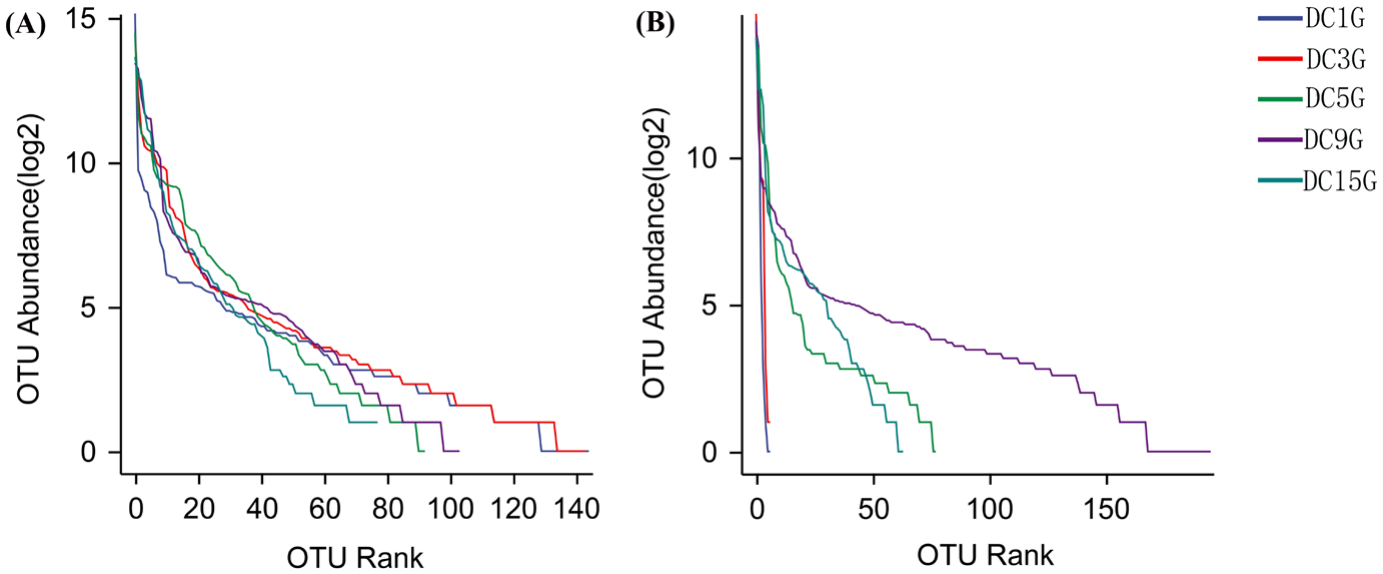
Rank abundance curve bacteria (A) and fungi (B).

The Rank‐Abundance curve in Figure 2 illustrates the abundance and uniformity of microorganism distribution during *Douchi* fermentation. The shape of the curve reflected the evenness of species composition, while the length on the horizontal axis indicated species richness. In Figure 2A, the five curves representing bacterial communities display similar shapes, suggesting consistent evenness in bacterial species composition throughout fermentation. Notably, the curve was widest on the third day of fermentation, indicating the highest species richness at this stage. This finding suggested that the third day of fermentation is a critical period for bacterial diversity. The shapes of the fungal curves showed greater variation, indicating differences in species composition evenness. On the 1st and 3rd days, the curves were similar, reflecting comparable evenness but very low species richness. In contrast, on the 5th, 9th, and 15th days, the curves indicated similar evenness but increased species richness, especially on the 9th day, when the curve was the widest. This suggested that fungal diversity peaked around the 9th day of fermentation.

Illumina HTS technology was applied to samples DC1G, DC3G, DC5G, DC9G, and DC15G at the bacterial and fungal levels (Table 1). After sequencing, the data were screened, resulting in 379,335 effective sequences for bacteria and 606,993 sequences for fungi. The microbial coverage of the samples reached 99.9%, indicating comprehensive detection of the microbial communities, thus accurately representing the real microbial diversity in the *Douchi* samples. The Chao index, which estimates species richness, showed that the OTU count for bacteria was highest in the DC1G sample (145 OTUs) and lowest in the DC5G sample (93 OTUs). For fungi, the highest OUT number was observed in the DC9G sample (189 OTUs), whereas the lowest was in the DC15G sample (66 OTUs). These results suggest significant variability in microbial richness across different fermentation stages. The Shannon and Simpson indices, which reflect both richness and evenness of species, indicated that bacterial diversity and richness were highest in the DC9G sample, with Shannon and Simpson values of 3.43 and 0.85, respectively. In contrast, fungal diversity and richness were highest in the DC15G sample, with Shannon and Simpson values of 2.45 and 0.69, respectively.

**TABLE 1:** Statistics of bacterial and fungal α diversity indices in bacterial‐type *Douchi* samples.

	Sample	Number of valid sequences	Chao	Coverage	OUT	Shannon	Simpson
Bacteria	DC1G	93,185	151.629	0.999642	145	1.24	0.25
	DC3G	99,635	149.019	0.999678	144	2.98	0.71
	DC5G	60,871	93.191	0.999959	93	2.99	0.68
	DC9G	65,308	104.057	0.999373	103	3.43	0.85
	DC15G	60,336	78.075	0.99951	78	3.25	0.83
Fungi	DC1G	82,112	6.75	0.999959	6	1.01	0.49
	DC3G	80,878	6.9	0.999983	7	0.68	0.22
	DC5G	60,586	78.636	0.999913	78	2.22	0.68
	DC9G	217,715	200.132	0.999478	189	2.21	0.45
	DC15G	65,702	68.152	0.999854	66	2.45	0.69

Overall, these α diversity indices provide a detailed understanding of the microbial diversity in *Douchi* fermentation. The high coverage confirms that the sequencing depth was sufficient to capture the majority of microbial species present, whereas the Chao, Shannon, and Simpson indices reveal dynamic changes in microbial community structure and diversity throughout the fermentation process. These findings underscore the importance of specific fermentation stages in shaping the microbial composition and diversity in *Douchi*. Among them, the production of siloxane derivatives may be due to the degradation of silica gel in the fermentation cap during fermentation, and the siloxane derivatives are released and melted into tempeh, producing oxythane derivatives. Or a siloxane derivative released by the column itself during GC–MS detection.

### 3.2 OTU Statistics and Taxonomic Analysis

**FIGURE 3:**
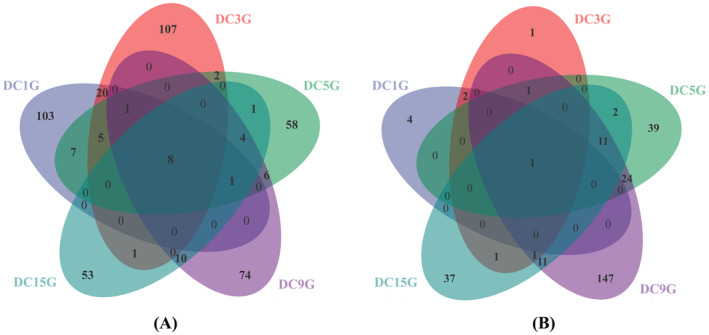
OTU distribution of bacteria (A) and fungi (B).

Sequences were clustered into groups based on similarity, with each group representing an OTU. Bioinformatics analysis was conducted on OTUs clustered at a 97% similarity level. Cluster analysis of 379,335 bacterial and 606,993 fungal valid sequences obtained from HTS yielded a total of 461 bacterial OTUs and 282 fungal OTUs. Venn diagrams based on the OTU counts are shown in Figure 3. The analysis revealed that bacterial types in this fermentation were more abundant than fungi types. The number of bacterial OTUs was lowest on day 15, whereas the highest counts were observed on days 1 and 2. Among fungal OTUs, sample DC9G had the highest number of OTUs and the largest OTU abundance, indicating significant fungal diversity at this fermentation stage. During prefermentation, the number of bacterial OTUs was higher than that of fungi OTUs. In postfermentation, both fungi and bacteria OTU counts initially increased and then decreased. Species diversity peaked on day 9, while the lowest diversity was recorded on day 15. On day 5, a decrease in bacterial OTUs and an increase in fungal OTUs were observed, likely due to the addition of salt, wine, and other condiments on day 3, which may have inhibited bacterial growth. As fermentation progressed, the number of strains increased, peaking on day 9, and then gradually decreased during the postripening stage. Additionally, eight bacterial OTUs and one fungal OTU persisted throughout the entire fermentation process, indicating that certain microorganisms remained active during *Douchi* fermentation. This persistence suggested a core microbiota that played a crucial role in the fermentation process.

### 3.3 Distribution Characteristics at the Microbial Phylum Level

**FIGURE 4:**
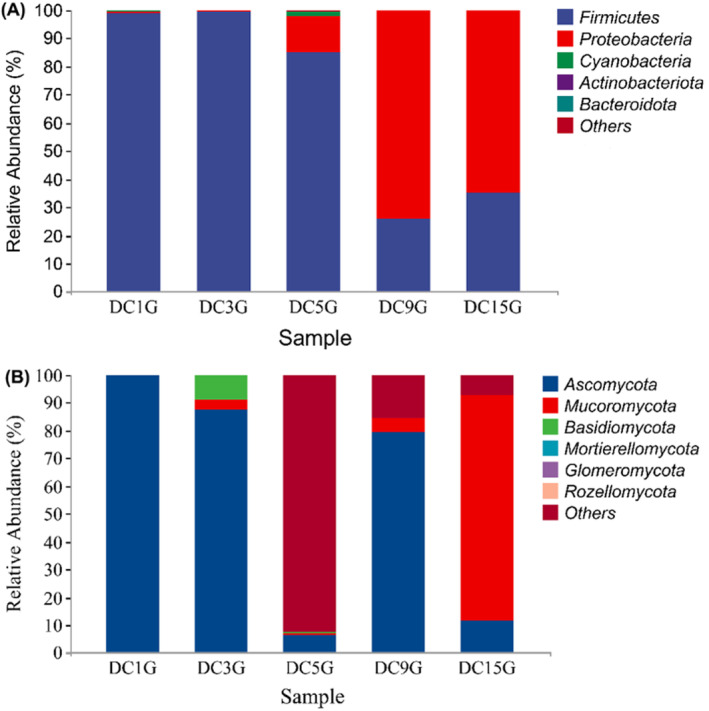
Comparison of the relative abundance of bacteria (A) and fungi (B) at the phylum level during *Douchi* fermentation.

As shown in Figure 4A, the analysis of the five bacterial samples revealed the presence of the following phyla: Firmicutes, Proteobacteria, Cyanobacteria, Actinobacteria, Bacteroidota, and some unclassified groups. At the fungal level, five phyla were identified: Ascomycota, Mucoromycota, Basidiomycota, MortierelLomycota, and Glomeromycota. The bacterial community structure varied among the five *Douchi* samples. Firmicutes and Proteobacteria were the dominant bacterial phyla, initially comprising approximately 85% of the bacterial community. However, the abundance of Firmicutes significantly reduced (*p <* 0.05) to only 30% by day 15. Conversely, the abundance of Proteobacteria increased significantly (*p <* 0.05) from 1% at the beginning to 65% during fermentation. This indicates a shift in dominant phyla as fermentation progressed, with Proteobacteria becoming more prevalent in the later stages of fermentation.

Among the fungal phyla (Figure 4B), Ascomycota was dominant on days 1, 3, and 9 of fermentation, with an average relative abundance of 85%. However, the content of Ascomycota decreased significantly in the later stages of fermentation. In contrast, Mucoromycota content gradually increased from 0% to 81% during the fermentation process, becoming the dominant fungal phylum by the later stages. This shift indicates a transition in fungal community structure, with Mucoromycota emerging as the predominant phylum as fermentation progressed, while the abundance of Ascomycota declined.

The data illustrate significant changes in the microbial community composition at both the bacterial and the fungal phylum levels during *Douchi* fermentation. Initially, Firmicutes and Ascomycota were the dominant phyla, but their abundance decreased significantly over time. Proteobacteria and Mucoromycota became the predominant phyla in the later stages of fermentation. These findings highlight the dynamic nature of microbial succession during the fermentation process, with specific phyla becoming predominant at different stages. Understanding these shifts is essential for optimizing fermentation conditions and enhancing the quality of *Douchi*.

### 3.4 Distribution Characteristics at the Microbial Genus Level

**FIGURE 5:**
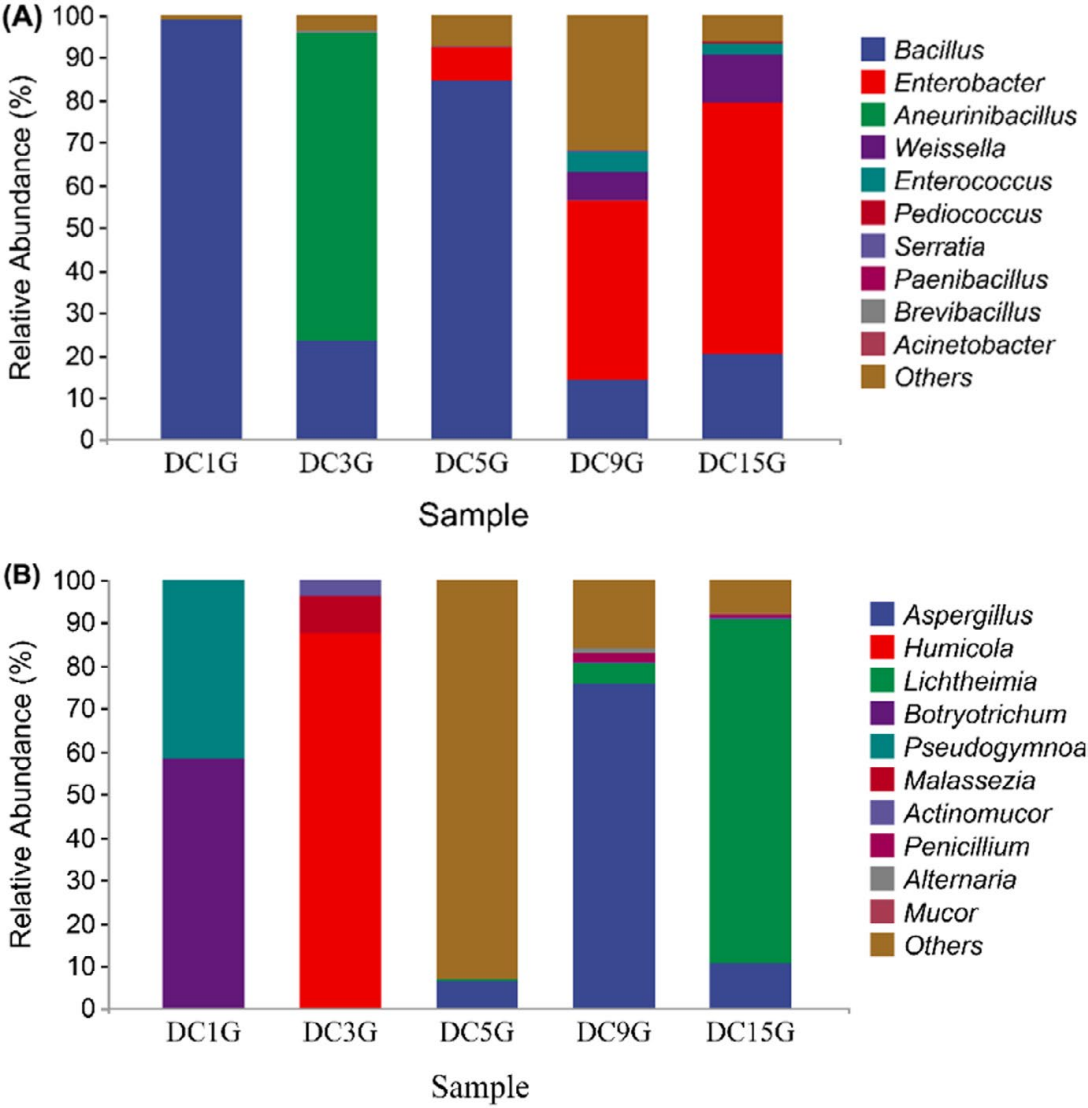
Comparison of the relative abundance of bacteria (A) and fungi (B) at the genus level during the fermentation process of *Douchi*.

Based on the taxonomic annotation results at the genus level, five dominant genera among the 11 identified in *Douchi* samples were plotted, as shown in Figure 5A. The main bacterial genera identified were *Bacillus*, *Enterobacter*, *Aneurinibacillus*, *Weissella*, and *Enterococcus*. Bacillus was the dominant bacterium, with its relative abundance being very high on days 1 and 5, reaching 98% on day 1 and 84% on day 5. Its content then gradually decreased to 14% on day 9 before slightly increasing to 20% on day 15. In contrast, the content of Enterobacter and Weissella increased over time, gradually becoming the dominant genera in the later stages of fermentation. The dominant fungal genera were *Aspergillus*, *Humicola*, *Lichtheimia*, and *Botryotrichum* (Figure 5B). Aspergillus was absent on the first and third days, with its relative abundance reaching 6% on day 5. By day 9, Aspergillus became the dominant genus with a content of 75%, but its abundance decreased to 10% by day 15, indicating suppression during the later stages of fermentation. Conversely, *Humicola* had a relative abundance of 87% on day 3 but was absent later, possibly due to the addition of salt and wine, which may have inhibited its growth (Yu et al. 2022). The abundance of *Lichtheimia* gradually increased from 0.6% on day 5 to 80% on day 15, becoming the dominant genus in the later stages of fermentation.

### 3.5 HS‐SPME‐GC–MS Identification of *Douchi* Flavor Components During Fermentation

Techniques for measuring volatile components in *Douchi* include HS‐SPME‐GC‐MS, GC‐O, and electronic nose technology (Jiang et al. 2020). Among these, HS‐SPME‐GC‐MS is the most commonly used for detecting volatile components, accurately quantifying substances, and exploring the volatile composition of *Douch* (Zhao et al. 2023). In this study, HS‐SPME‐GC‐MS was used to detect volatile components in naturally fermented bacterial‐type *Douchi* samples with a relative content ≥ 0.03% (Table 2).

**TABLE 2:** Comparison of volatile components during bacterial‐type *Douchi* fermentation.

Sequence	Name	Molecular formula	Relative content %				
			DC1Q	DC3Q	DC5Q	DC9Q	DC15Q
1	Silane, ethoxytriethyl	C8H20OSi	0.1	0.39			
2	Cyclotetrasiloxane, octamethyl	C8H24O4Si4	1.15	4.91	6.42	2.22	0.34
3	Decane, 2‐methyl	C11H24	0.03		0.07	0.03	
4	Hydroperoxide, 1‐methylhexyl	C7H16O2	0.01		0.03	0.29	
5	Cyclopentasiloxane, decamethyl	C10H30O5Si5	14.22	0.03	17.79	9.86	0.03
6	Dodecane	C12H26		0.09		0.14	0.03
7	1,3‐Dithiane	C4H8S2				3.04	
8	Oxirane, 2‐butyl‐3‐methyl‐, cis	C7H14O		0.84	0.16		
9	3‐Isopropoxy‐1,1,1,7,7,7‐hexamethyl‐3,5,5‐tris(trimethylsiloxy)tetrasiloxane	C18H52O7Si7		1.01	0.52		
10	Z,Z,Z‐4,6,9‐Nonadecatriene	C19H34				0.40	
11	Cyclohexane, 1‐ethenyl‐1‐methyl‐2,4‐bis(1‐methylethenyl)	C15H24			0.03	0.06	0.03
12	1,2‐Dioxetane, 3,4,4‐trimethyl‐3‐[[(trimethylsilyl)oxy]methyl]	C9H20O3Si				0.12	
13	Z,Z,Z‐1,4,6,9‐Nonadecatetraene	C19H32			0.02		0.03
14	Heptadecane, 2,6‐dimethyl	C19H40				0.03	0.07
15	Octa‐2,4,6‐triene	C8H12				0.14	
16	2‐Methyl‐1‐tetradecene	C15H30				0.03	0.14
17	Benzene, 1‐(1,5‐dimethyl‐4‐hexenyl)‐4‐methyl	C15H22			0.17	0.03	0.65
18	trans‐à‐Bergamotene	C15H24				0.27	0.02
19	2‐amino‐1‐(3,4‐methylenedioxyphenyl)‐butane	C11H15NO2		0.06			
20	Octadecane, 3‐ethyl‐5‐(2‐ethylbutyl)—	C26H54		0.07			
21	2,3‐Epoxybutane	C4H8O					0.15
22	1,3,6,10‐Dodecatetraene, 3,7,11‐trimethyl‐, (Z,E)	C15H24					0.15
23	Dodecane, 5‐methyl	C13H28			0.09		0.02
24	Tridecane, 2‐methyl	C14H30					0.93
25	1,3‐Cyclohexadiene, 5‐(1,5‐dimethyl‐4‐hexenyl)‐2‐methyl‐, [S‐(R*,S*)]	C15H24			0.46	0.23	1.45
26	Pinene	C10H16			3.55	1.11	0.29
27	ç‐Terpinene	C10H16			0.89	0.53	0.3
28	Cyclohexasiloxane, dodecamethyl	C12H36O6Si6	3.33	10.33	5.50	3.03	
29	Cyclohexene, 3‐(1,5‐dimethyl‐4‐hexenyl)‐6‐methylene‐, [S‐(R*,S*)]	C15H24			0.13		0.39
30	Hexasiloxane, tetradecamethyl—	C14H42O5Si6		0.05			
31	Hydroperoxide, 1‐methylbutyl	C5H12O2				0.11	0.14
32	2,3‐Epoxybutane	C4H8O				0.11	
33	9‐methylheptadecane	C18H38		0.09		0.14	
34	Methane, nitroso	CH3NO			13.61	13.90	9.45
35	Ethanolamine	C2H7NO	16.89	1.46			0.22
36	2,3‐Butanediol, [S‐(R*,R*)]	C4H10O2	14.30			3.48	11.0
37	2,3‐Butanediol, [R‐(R*,R*)]	C4H10O2	0.07			5.80	0.09
38	Ethanol	C2H6O			0.78	0.59	
39	Ethanol, 2‐nitro	C2H5NO3			0.06		0.35
40	2‐Heptanol	C7H16O			0.18	0.52	3.27
41	1‐Butanol, 3‐methyl	C5H12O					0.54
42	1‐Octen‐3‐ol	C8H16O				0.57	1.31
43	(2S,3S)‐(−)‐3‐Propyloxiranemethanol	C6H12O2		0.03			
44	2‐Heptanol, 4‐methyl	C8H18O		0.24		0.3	0.6
45	Bicyclo[2.2.1]heptan‐2‐ol, 1,7,7‐trimethyl‐, (1S‐endo)	C10H18O			0.29	0.21	0.21
46	2‐Propanol, 1‐(1‐methylethoxy)	C6H14O2					9.42
47	Eucalyptol	C10H18O			0.03	1.09	0.03
48	(3‐Methyl‐oxiran‐2‐yl)‐methanol	C4H8O2				0.03	0.03
49	1‐Deoxy‐d‐mannitol	C6H14O5					0.12
50	2‐Pentanol, 3‐methyl—	C6H14O				0.25	0.29
51	Bicyclo[2.1.1]hexan‐2‐ol, 2‐ethenyl—	C8H12O		0.38			
52	Butanal, 3‐hydroxy	C4H8O2	0.03		0.06		
53	Butanal, 3‐methyl	C5H10O		1.69			
54	Benzeneacetaldehyde	C8H8O				0.15	
55	Benzaldehyde	C7H6O		9.98	0.07		
56	Benzaldehyde, 2,5‐bis[(trimethylsilyl)oxy]	C13H22O3Si2		0.23			
57	l‐Gala‐l‐ido‐octose	C8H16O8		0.10	0.10	0.16	
58	2‐Butanone, 3‐hydroxy	C4H8O2	43.84				
59	2‐Heptanone	C7H14O	0.98	2.62	2.14		
60	2‐Pentanone, 3‐methyl	C6H12O		3.51	0.92		
61	2‐Nonanone	C9H18O		0.46	0.44	0.18	0.03
62	2‐Decanone	C10H20O		0.16			
63	2‐Heptanone, 6‐methyl	C8H16O	0.07	3.39	1.47	1.47	0.33
64	3‐(Hydroxy‐phenyl‐methyl)‐2,3‐dimethyl‐octan‐4‐one	C17H26O2			2.81		
65	2‐Dodecanone	C12H24O			0.05	0.03	
66	3‐Hexanone, 4‐methyl	C7H14O		5.58	0.28		
67	N‐Acetyl‐L‐alanine	C5H9NO3	3.3				
68	Propanoic acid, 2‐methyl	C4H8O2		1.81		1.85	0.08
69	Butanoic acid, 2‐methyl	C5H10O2	0.03			2.27	0.18
70	Malonamic acid	C7H14O	0.87	2.36			
71	Creatine	C4H9N3O2		1.79			
72	Hexanoic acid	C6H12O2		0.16		0.06	1.03
73	Acetic acid	C2H4O2				2.23	0.32
74	4‐Pyridinepropanoic acid, à‐amino‐á‐hydroxy‐, [R‐(R*,S*)]	C8H10N2O3			0.15		
75	dl‐Allo‐cystathionine	C7H14N2O4S					1.47
76	Pterin‐6‐carboxylic acid	C7H5N5O3		29.67	0.03		
77	á‐Hydroxypyruvic acid, trimethylsilyl ether, trimethylsilyl ester	C9H20O4Si2	0.13		0.05		0.03
78	12,15‐Octadecadiynoic acid, methyl ester	C19H30O2			0.04		0.03
79	Ethanedioic acid, bis (trimethylsilyl) ester	C8H18O4Si2				0.27	0.18
80	Butanoic acid, 2‐methyl‐, ethyl ester	C7H14O2			0.07	0.35	0.99
81	Pentanoic acid, 4‐methyl‐, ethyl ester	C8H16O2			0.05		0.09
82	Pentanoic acid, octyl ester	C13H26O2					0.16
83	Nonanoic acid, ethyl ester	C11H22O2				0.14	0.04
84	Heptanoic acid, ethyl ester	C9H18O2				0.26	33.94
85	Ethyl Acetate	C4H8O2				1.09	
86	N‐[1‐(Benzylamino)‐2,2,2‐trifluoro‐1‐(trifluoromethyl)ethyl]butyramide	C14H16F6N2O	0.11				
87	Pyridine, 2,3,4,5‐tetrahydro	C5H9N		2.80			
88	Pyrimidine, 4‐methyl	C5H6N2		0.33	0.12		
89	p‐Xylene	C8H10			0.57		
90	o‐Cymene	C10H14			0.12		
91	Cyclopenta[c]furo[3′,2′:4,5]furo[2,3‐h][1]benzopyran−11(1H)‐one, 2,3,6a,9a‐tetrahydro‐1,3‐dihydroxy‐4‐methoxy	C17H14O7				0.04	0.43
92	5‐Amino‐1‐benzoyl‐1H‐pyrazole‐3,4‐dicarbonitrile	C12H7N5O		0.40	0.03		
93	3‐Hydroxybutanamide, N‐phenylmethoxy	C11H15NO3				0.30	
94	Pyrazine, 2,5‐dimethyl	C6H8N2		5.06	3.16	0.04	0.12
95	Inabenfide	C19H15ClN2O2		0.90			
96	Phenelzine	C8H12N2	0.07				
97	Diallyl disulphide	C6H10S2			26.68	37.81	6.95
98	Diallyl sulfide	C6H10S				0.86	0.20
99	3,4‐Dimethyl‐2‐prop‐2‐enyl‐2,5‐dihydrothiophene 1,1‐dioxide	C9H14O2S				0.33	
100	Trisulfide, di‐2‐propenyl	C6H10S3			0.24	0.02	2.77
101	Sulfide, allyl methyl	C4H8S				0.09	0.25
102	Disulfide, methyl 1‐propenyl	C4H8S2				0.14	0.42
103	Oxirane, [(1‐methylethoxy)methyl]	C6H12O2	0.26				
104	Bicyclo[3.1.0]hex‐2‐ene, 4‐methyl‐1‐(1‐methylethyl)	C10H16			7.41		
105	á‐Bisabolene	C15H24			0.16	0.05	
106	2‐Methyl‐l‐methylmannopyranoside	C7H14O5				1.2	
107	Propanamide, 2‐hydroxy	C3H7NO2			0.26	0.08	0.91
108	Ala‐Gly	C5H10N2O3		6.45	0.80	0.2	0.58
109	D‐(−)‐Tagatose	C6H12O6		0.11			
110	Propane, 2‐(ethenyloxy)	C5H10O		0.15	0.11		

Table 2 shows that 110 flavor substances were identified by HS‐SPME‐GC–MS analysis of *Douchi* samples, including 34 hydrocarbons, 17 alcohols, 11 heterocyclic compounds, 10 acids, 9 ketones, 9 esters, 6 aldehydes, 6 sulfur‐containing compounds, and 8 other compounds. The total number of detected compounds was 20 in DC1Q, 39 in DC3Q, 49 in DC5Q, 59 in DC9Q, and 56 in DC15Q. The types of hydrocarbons, alcohols, esters, and sulfur‐containing compounds positively correlated with the number of fermentation days, with more extended fermentation resulting in a higher variety. Compounds with a total content higher than 10% included octamethylcyclotetrasiloxane, decamethylcyclopentasiloxane, dodecamethylcyclohexasiloxane, ethanolamine, 2,3‐butanediol, benzoin, 3‐hydroxy‐2‐butanone, pterin‐6‐carboxylic acid, ethyl heptanoate, and diallyl disulfide.

Nie et al. (2022) conducted GC/MS analysis on naturally fermented dried *Douchi* from Zunyi, detecting 91 volatile substances, with acids accounting for the highest proportion at 44.529%. In this study, hydrocarbons accounted for the largest proportion at 27.29%, followed by alcohols at 15.01%, ketones at 14.15%, acids at 9.90%, esters at 7.58%, and aldehydes at 2.51%. Hydrocarbons showed an upward trend in the first 3 days, peaking at 49.43% on day 3, and gradually decreasing thereafter. Alcohols were 31% on day 1, reduced to 2% on day 5, and increased to 27% on day 15, showing an opposite trend to hydrocarbons. Ketones constituted 44% on day 1 and gradually decreased throughout fermentation. Esters and sulfur‐containing compounds reached their highest levels on day 15. Overall, flavor compound content gradually increased, peaking on day 9, coinciding with the highest OTU counts of microbial flora, indicating a correlation between microbial flora and flavor substance types.

### 3.6 Correlation Analysis Between *Douchi* Microbial Diversity and Fragrance Components

The main methods of the correlation analysis between *Douchi* microbial diversity and volatile components include Pearson, Spearman, and bidirectional orthogonal partial least squares (O2PLS) (Zhang et al. 2022). In this study, Spearman correlation analysis was used to establish potential associations between flavor substances and the structure of bacterial‐type *Douchi* microflora. The dominant genera from HTS and 20 flavor substances (content > 1%) identified by HS‐SPME‐GC‐MS were selected, and a correlation heat map was drawn.

**FIGURE 6:**
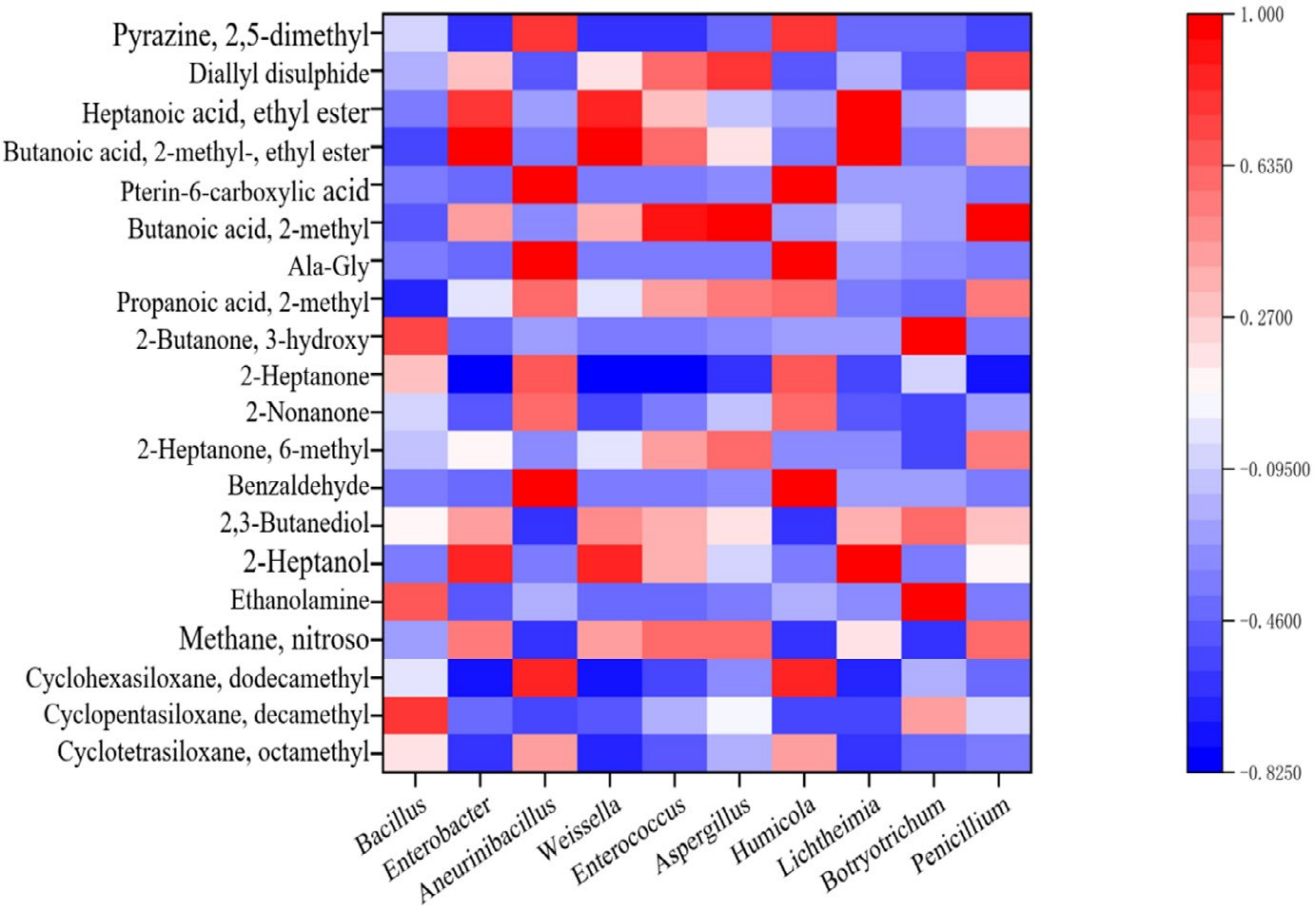
Heat map of the correlation between microbial genera and volatile substances in *Douchi*.

In Figure 6, red represents a positive correlation, blue represents a negative correlation, and darker colors indicate stronger correlations. The changes in many characteristic flavor compounds during bacterial‐type *Douchi* fermentation were related to microbial activity, with specific strains influencing flavor development. Benzoinal, L‐alanylglycine, pterre‐6‐carboxylic acid, and 2,5‐dimethylpyrazine showed a strong positive correlation with *Aneurinibacillus* and *Humicola*. 2‐Heptanol, methyl 2‐methylvalerate, and ethyl heptanoate were positively correlated with *Enterobacter*, *Weissella*, and *Lichtheimia*. Ethanolamine and 3‐hydroxy‐2‐butanone were positively correlated with *Botryotrichum*, while 2‐Methylbutyric acid and diallyl disulfide were positively correlated with *Enterococcus*, *Aspergillus*, and *Penicillium*. The genera *Enterobacter*, *Weissella*, *Lichtheimia*, *Aneurinibacillus*, *Humicola*, *Enterococcus*, and *Aspergillus* may contribute similar to flavor formation. Zhang et al. (2023) used the partial least squares method to analyze the correlation between bacterial microorganisms and flavor substances in *Aspergillus Douchi* samples, identifying 57 genera (VIP (pred) > 1.0) with significant effects on flavor. They found that *Peptostreptococcus* and *Actinomycetaceae* were positively correlated with many volatile substances, promoting flavor formation in *Douchi*. Mi et al. (2019) showed that microorganisms such as *Lactobacillus* and *Weissiella* might affect alcohols and esters, consistent with our findings. Tian et al. (2023) studied the influence of bacilli on alcohol production, aligning with our results. These results highlight the dynamic interactions between microbial communities and volatile compounds throughout *Douchi* fermentation. Understanding these correlations is crucial for optimizing fermentation processes and enhancing the flavor profile of *Douchi*.

## 4 Conclusion

Analyzing the relationship between bacterial‐type *Douchi* microorganisms and flavor compounds is crucial for identifying functional flavor‐producing strains and the flavor characteristics of controlled fermented foods. This study reveals differences in microbial diversity and volatile compounds throughout the fermentation process of bacterial‐type *Douchi*. Using high‐throughput sequencing (HTS) and headspace‐solid phase microextraction gas chromatography–mass spectrometry (HS‐SPME‐GC–MS), this study analyzed the microbial flora structure and volatile compounds, and establishing correlation between volatile flavors and dominant bacteria through Spearman analysis. The findings of this study contribute to understanding the mechanism of flavor formation through microbiota community structure. These findings provide an important reference for understanding the correlation between the microbiota community structure and the characteristic flavor of bacterial‐type *Douchi*, offering a theoretical basis for improving the flavor and optimizing fermentation processes to enhance *Douchi's* sensory qualities.

## Author Contributions

**Aiguo Luo:** funding acquisition (equal), investigation (equal), methodology (equal), project administration (equal), resources (equal), writing – original draft (equal). **Tiantian Liu:** investigation (equal), methodology (equal), writing – original draft (equal). **Shengli Shi:** data curation (equal), formal analysis (equal), investigation (equal). **Xiaoxia Liu:** data curation (equal), formal analysis (equal). **Xiaoli Shi:** project administration (equal), software (equal), validation (equal). **Bianfang Hu:** conceptualization (equal), project administration (equal), writing – review and editing (equal).

## Conflicts of Interest

The authors declare no conflicts of interest.

## Data Availability

The data that support the findings of this study are available from the corresponding author, Aiguo Luo, upon reasonable request.

## References

[fsn34660-bib-0001] Anderson, M. J. , K. E. Ellingsen , and B. H. McArdle . 2006. “Multivariate Dispersion as a Measure of Beta Diversity.” Ecology Letters 9, no. 6: 683–693. 10.1111/j.1461-0248.2006.00926.x. 16706913

[fsn34660-bib-0002] Anderson, M. J. , and D. C. I. Walsh . 2013. “PERMANOVA, ANOSIM, and the Mantel Test in the Face of Heterogeneous Dispersions: What Null Hypothesis Are You Testing.” Ecological Monographs 83, no. 4: 557–574. 10.1890/12-2010.1.

[fsn34660-bib-0003] Anderson, M. J. , and T. J. Willis . 2003. “Canonical Analysis of Principal Coordinates: A Useful Method of Constrained Ordination for Ecology.” Ecology 84, no. 2: 511–525. 10.1890/0012-9658(2003)084[0511:CAOPCA]2.0.CO;2.

[fsn34660-bib-0004] Edgar, R. C. 2010. “Search and Clustering Orders of Magnitude Faster Than BLAST.” Bioinformatics 26, no. 19: 2460–2461. 10.1093/bioinformatics/btq461. 20709691

[fsn34660-bib-0005] Geng, Y. H. , and G. J. Li . 2005. “GC/MS Analysis of Fatty Acid Components in *Douchi* .” Chinese Brewing 149, no. 8: 51–52.

[fsn34660-bib-0006] Huang, X. R. , Y. Guo , Z. J. Li , J. X. Zeng , and Y. J. Wu . 2020. “Dynamic Monitoring of Bacterial Communities in Naturally Fermented Water *Douchi* .” Chinese Seasonings 45, no. 6: 103–106. 10.3969/j.issn.1000-9973.2020.06.022.

[fsn34660-bib-0007] Jiang, L. W. , Y. H. Xie , P. Li , L. L. Chen , H. L. Zhou , and Y. Chen . 2020. “HS‐SPME/GC‐MS and Electronic Sensory Technology Analysis of Flavor Quality During the Fermentation Process of Mucor‐Type *Douchi* .” Journal of Nuclear Agriculture 34, no. 7: 1497–1506. 10.11869/j.issn.100-8551.2020.07.1497.

[fsn34660-bib-0008] Li, A. , X. Feng , G. Yang , et al. 2024. “Impact of Aroma‐Enhancing Microorganisms on Aroma Attributes of Industrial *Douchi*: An Integrated Analysis Using E‐Nose, GC‐IMS, GC‐MS, and Descriptive Sensory Evaluation.” Food Research International 182: 114181. 10.1016/j.foodres.2024.114181. 38519190

[fsn34660-bib-0009] Li, W. , S. B. Luo , Z. R. Qiu , D. L. Lai , H. W. Wang , and H. Y. Suo . 2020. “Microbial Community Structure and Dynamic Succession in Traditional Fermentation of Mucor‐Type Yongchuan *Douchi* .” Food and Fermentation Industries 46, no. 23: 60–67. 10.13995/j.cnki.11-1802/ts.024566.

[fsn34660-bib-0010] Li, X. R. , X. F. Liu , and H. Y. Zhang . 2014. “Using High‐Throughput Sequencing to Analyze the Diversity of *Douchi* Microbial Communities in Two Regions of Yunnan.” Modern Food Technology 30, no. 12: 61–67. 10.13982/j.mfst.1673-9078.2014.12.011.

[fsn34660-bib-0011] Liang, Y. X. , L. Zhang , F. H. Gao , et al. 2017. “Research Progress on Production Technology and Physiological Active Ingredients of Water *Douchi* .” Southern Agriculture 11, no. 34: 36–38. 10.19415/j.cnki.1673-890x.2017.34.009.

[fsn34660-bib-0012] Liu, L. B. , X. Q. Chen , L. L. Hao , et al. 2020. “Traditional Fermented Soybean Products: Processing, Flavor Formation, Nutritional and Biological Activities.” Critical Reviews in Food Science and Nutrition 62, no. 7: 1971–1989. 10.1080/10408398.2020.1848792. 33226273

[fsn34660-bib-0014] Mi, R. F. , X. Chen , S. Y. Xiong , et al. 2019. “Analysis of Bacterial Community Diversity and Flavor Quality in Traditional Naturally Fermented Sour Meat.” Food Science 40, no. 2: 85–92. 10.7506/spkx1002-6630-20180521-282.

[fsn34660-bib-0015] Nie, Q. L. , X. J. Wang , L. X. Liu , Y. H. Chen , H. H. Bao , and C. X. He . 2022. “Analysis of Microbial Community Structure and Flavor Quality of Zunyi Naturally Fermented Dry Lobster Sauce.” Food Science 43, no. 14: 158–164. 10.7506/spkx1002-6630-20210726-299.

[fsn34660-bib-0016] Tian, Y. X. , Y. C. Mu , W. Su , and X. D. Yin . 2023. “Correlation Between Microorganisms and Volatile Flavor Compounds Stage of Chopped Pepper With Ginger Shreds During Post‐Ripening.” Food Science 44, no. 4: 185–193. 10.7506/spkx1002-6630-20220316-187.

[fsn34660-bib-0017] Wang, Y. , F. Xiang , Z. Zhang , Q. Hou , and Z. Guo . 2021. “Characterization of Bacterial Community and Flavor Differences of Different Types of *Douchi* .” Food Science & Nutrition 9, no. 7: 3460–3469. 10.1002/fsn3.2280. 34262706 PMC8269581

[fsn34660-bib-0018] Xu, J. W. , W. M. Wang , H. S. Shi , et al. 2022. “Research Progress on the Microbiome and Functional Components of *Douchi* and Light *Douchi* .” China Brewing 41, no. 6: 18–23. 10.11882/j.issn.0254-5071.2022.06.004.

[fsn34660-bib-0019] Yang, Z. B. , X. Q. Tan , and C. Q. Zhou . 2023. “Comparative Analysis of the Food Safety and Flavor Characteristics of Bacterial Fermented *Douchi* in Chongqing Guizhou Region.” Food and Fermentation Industry 49, no. 5: 124–133. 10.13995/j.cnki.11-1802/ts.031869.

[fsn34660-bib-0021] Yu, S. J. , L. Jiang , X. Y. Chai , X. Y. Xu , and Z. Z. Tu . 2022. “Analysis of Bacterial Diversity of Several Traditional Fermented *Douchi* .” Journal of Anqing Normal University 28, no. 2: 78–84. 10.13995/j.cnki.11-1802/ts.027606.

[fsn34660-bib-0022] Zhang, Y. C. , X. J. Tang , X. N. Lin , et al. 2023. “Research Progress on Detection Technology and Correlation Analysis of Microbial Diversity and Volatile Components of Tempeh.” China Condiment 48, no. 1: 210. 10.3969/j.issn.1000-9973.2023.01.038.

[fsn34660-bib-0023] Zhang, Y. Z. , X. N. Lin , Y. Q. Ji , et al. 2022. “Characterization and Correlation of Dominant Bacteria and Volatile Compounds in Post‐Fermentation Process of Ba‐Bao *Douchi* .” Food Research International 160: 111688. 10.1016/j.foodres.2022.111688. 36076449

[fsn34660-bib-0024] Zhao, H. , J. Xu , R. Wang , X. Liu , X. Peng , and S. Guo . 2023. “Succession and Diversity of Microbial Flora During the Fermentation of *Douchi* and Their Effects on the Formation of Characteristic Aroma.” Food 12, no. 2: 329. 10.3390/foods12020329. PMC9857697 36673421

